# Evaluation of an artificial intelligence-based system for echocardiographic estimation of right atrial pressure

**DOI:** 10.1007/s10554-023-02941-8

**Published:** 2023-09-08

**Authors:** Ghada Zamzmi, Li-Yueh Hsu, Sivaramakrishnan Rajaraman, Wen Li, Vandana Sachdev, Sameer Antani

**Affiliations:** 1 National Library of Medicine, National Institutes of Health, 8600 Rockville Pike, Bethesda, MD 20894 USA; 2 Clinical Center, National Institutes of Health, 10 Center Dr, Bethesda, MD 20892 USA; 3 National Heart, Lung, and Blood Institute, National Institutes of Health, 9000 Rockville Pike, Bethesda, MD 20892 USA

**Keywords:** Echocardiography, Machine learning, Artificial intelligence, Right Atrial Pressure, Inferior Vena Cava, Collapsibility Analysis

## Abstract

Current noninvasive estimation of right atrial pressure (RAP) by inferior vena cava (IVC) measurement during echocardiography may have significant inter-rater variability due to different levels of observers’ experience. Therefore, there is a need to develop new approaches to decrease the variability of IVC analysis and RAP estimation. This study aims to develop a fully automated artificial intelligence (AI)-based system for automated IVC analysis and RAP estimation. We presented a multi-stage AI system to identify the IVC view, select good quality images, delineate the IVC region and quantify its thickness, enabling temporal tracking of its diameter and collapsibility changes. The automated system was trained and tested on expert manual IVC and RAP reference measurements obtained from 255 patients during routine clinical workflow. The performance was evaluated using Pearson correlation and Bland-Altman analysis for IVC values, as well as macro accuracy and chi-square test for RAP values. Our results show an excellent agreement (r=0.96) between automatically computed versus manually measured IVC values, and Bland-Altman analysis showed a small bias of $-$0.33 mm. Further, there is an excellent agreement ($(p<0.01$) between automatically estimated versus manually derived RAP values with a macro accuracy of 0.85. The proposed AI-based system accurately quantified IVC diameter, collapsibility index, both are used for RAP estimation. This automated system could serve as a paradigm to perform IVC analysis in routine echocardiography and support various cardiac diagnostic applications.

## Introduction

Echocardiography offers several advantages over other imaging modalities for diagnosing inferior vena cava (IVC) abnormalities. Examples of these advantages include high temporal resolution, non-invasiveness, low cost, and portability [1]. However, manual echocardiography analysis by a trained sonographer is time-consuming, costly, and may exhibit poor reproducibility [1, 2]. The utilization of automated analysis can streamline clinical workflows, provide consistent results, and might subsequently enhance clinical decision making [1, 2].

IVC is responsible for circulating deoxygenated blood from the lower extremities and abdomen back to the right atrium. Studies [3, 4] have reported that the diameter of IVC (dIVC) and its change with inspiration (a.k.a., IVC collapsibility) can be non-invasively captured using ultrasound imaging and used to determine the fluid status in critically ill patients and acute heart failure (HF) conditions, and this is now a routine part of clinical echo exams. The collapsibility index of the inferior vena cava (cIVC) is visually estimated based on the changes in IVC diameter with inspiration. The current practice of measuring dIVC, cIVC, and estimating RAP involves several steps. First, a sonographer needs to manually select a high visual quality subcostal long-axis view of the IVC from an echocardiography study that may contain over a hundred views. Then, the dIVC is measured perpendicular to the long axis of the IVC within 1.0 to 2.0 cms (cm) of the cavo-atrial junction [4]. The cIVC is measured as the difference between the maximum and minimum IVC diameters during inspiration. Finally, the measured dIVC and collapsibility can be used to readily estimate right atrial pressure (RAP) using the American Society of Echocardiography (ASE) guidelines or other guidelines [4, 5].

Although this approach for estimating RAP based on echocardiographic IVC is considered the current standard, the manual calculation of RAP [6–8] may have low reproducibility and weak correlation to actual RAP values. For example, Magnino et al. [6] found that the r-squared values for IVC diameter and collapsibility were 0.19 or lower, and the actual values were within 2.5 mmHg only 34% of the time. Such inaccuracy may lead to an overestimation of pulmonary pressure, which could result in inappropriate diuretic treatment choices, and ultimately lead to increased uncertainty in clinical outcomes. Therefore, there is a need to develop new approaches for more accurate and objective IVC analysis and RAP estimation. In this work, we hypothesize that advances in machine learning (ML) and artificial intelligence (AI) techniques enable the development of a novel fully automated, reproducible, and scalable pipeline for echocardiographic dIVC and cIVC analysis, and RAP estimation that could be used by experts and non-experts alike, in both high- and low-resource primary care.

ML algorithms have been widely used for the automated analysis and interpretation of echocardiography [2] as well as automated quantification of cardiac measurements including ejection fraction [9, 10], thickness of left ventricle (LV) and surrounding walls [11, 12], LV strain [13], and doppler velocities [14]. Despite the clinical significance of IVC collapsibility analysis and RAP estimation, only a handful of studies [15–17] proposed automated solutions. For example, Mesin et al. [17] proposed a semi-automated method which uses a support vector machine (SVM) for RAP classification. The proposed method achieved 71% accuracy, which is higher than manual estimation using published guidelines (61% accuracy). Instead of using traditional ML methods, recent studies [15, 16] employed advanced deep learning (DL) methods such as long short-term memory (LSTM) for predicting fluid responsiveness in critically ill patients based on the analysis of IVC collapsibility. Other methods for automated IVC analysis can be found in [8, 18, 19].

Although the automated analysis of IVC and RAP has been explored, current methods (1) are applied directly to a manually pre-selected video or single-frame image from the echo video containing the IVC view; (2) have large and complex models that limit the usage on hand-held devices; and, (3) are designed for a “closed-world” environment where the training data is fixed with no opportunity for the AI to learn new knowledge, which limits their usability and generalizability in real-world clinical settings. To mitigate these challenges, our work presents a multi-stage AI system that can estimate dIVC, cIVC, and RAP. The proposed system employs a lightweight and open-world ML architecture to rapidly generate dIVC, cIVC, and RAP values. The lightweight feature would facilitate its integration into handheld devices, which can enhance accessibility. The open-world feature makes the system more robust in detecting and learning new, unpredictable cases or scenarios in real-world clinical settings. We believe that our system is the first one that has these capabilities to estimate RAP reliably and automatically. We evaluate the performance of the proposed system on clinical routine echocardiograms and validate its accuracy against measurements made by human experts. Finally, we describe some limitations as well as potential applications of the proposed system.

**Fig. 1:**
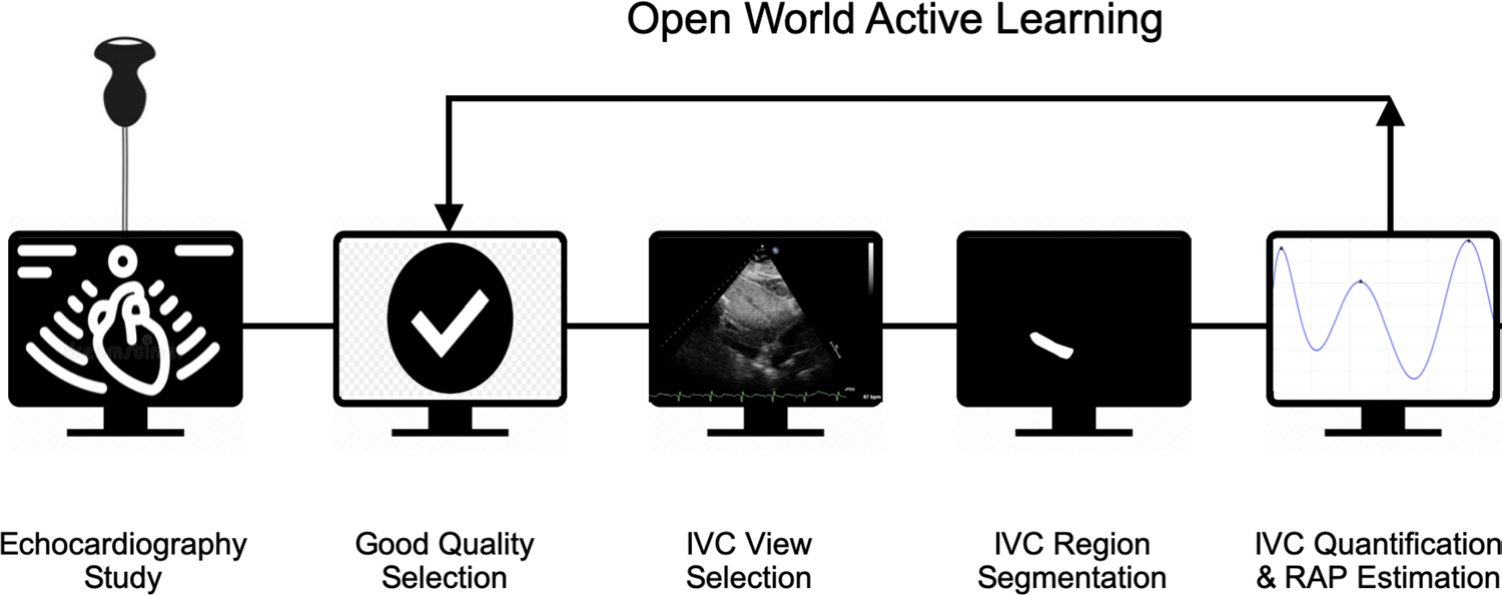
Automated pipeline for echocardiography assessment of dIVC, cIVC, and RAP estimation

## Materials and methods

All echocardiography exams were performed at the Clinical Center of the National Institutes of Health (NIH). This project was reviewed by the NIH Office of Human Subjects Research Protections, which determined that the activities proposed did not require IRB review or approval because the project does not qualify as human subjects research (45 CFR 46.102) as defined in the federal regulations. De-identified echo videos/images of 255 adult participants were included in this study.

### Echocardiography dataset

Each echocardiogram study consists of a collection of videos and still images showing various cardiac views including the parasternal long axis view (PLAX), Doppler, and apical four-chamber view (A4C) in addition to the IVC subcostal view. These were acquired using diverse echocardiography devices including iE33, GE E9, and GE Vivid E95. The manual measurements of IVC diameter were provided by board-certified echocardiographers following conventional methodology in current clinical practice.

For all downstream analyses, DICOM formatted videos were converted into multidimensional numeric arrays of pixel intensities. The acquired IVC videos have a spatial resolution of $800 \times 600$ pixels. The individual dimensions of the arrays represent time, x and y coordinates in space, and additional dimensions (channels) to enable the encoding of color information. We divided the entire dataset (n = 255) into a training set ($\approx$ 70%, n = 177 patients) and a hold-out testing set ($\approx$ 30%, n = 78 patients). The training set was further divided into training and validation using 10 folds cross validation. The training set was used for the main tasks of our pipeline: (1) IVC view classification and quality assessment; (2) IVC segmentation; and (3) dIVC and cIVC quantification.

### Automated pipeline for echocardiography IVC collapsibility and RAP estimation

The proposed pipeline is divided into multiple stages that are depicted in Fig. 1. This pipeline starts by performing image quality assessment and subcostal IVC view retrieval, and then followed by region segmentation, quantification, collapsibility analysis, and RAP estimation. The image analysis algorithms for each of the individual stages in the pipeline are described in the following subsections.

#### Image analysis: quality assessment & view retrieval

The image retrieval algorithm retrieves a specific view with acceptable quality. Our algorithm utilizes a lightweight model with a shared encoder and two “heads”. This term is derived from the analogy that the network shares common weights but like twins whose bodies are conjoined at birth and can appear to have two heads, each head can concurrently compute a separate but related input (same input image, two different tasks). In our algorithm, the first head is used for the quality assessment task and the second head is used for the view classification task. The view classification head detects an IVC view from a given echo study while the quality assessment head labels a given IVC view as good quality or bad quality. Both heads are configured to work in parallel, which can enhance the efficiency of the image retrieval component. The entire network with the shared encoder and two heads has a small size and high inference speed (0.22 s), enabling its use in resource-limited and handled devices. Further discussion about this two-head model along with a visualization is provided in Appendix A.

Conventional machine learning models require samples of a specific set of classes (e.g., IVC view vs. non-IVC view) to be available during training. This assumption, which is known as a closed-world assumption, may be too strict for real-world environments that are open and often have unseen examples. To overcome this challenge, our classification algorithm is designed to run efficiently in open-world clinical settings. It utilizes an OpenMax function [20] instead of Softmax function. The OpenMax function can label new (previously unseen) classes of images as “unknown”; a critical limitation of the Softmax function is that it strictly labels a new class as one of the known classes. Further details on OpenMax can be found in [21] and are provided in Appendix B.

The significance of open-world active learning can be demonstrated in several IVC applications. One possible application would be to group rare cases of IVC morphology that might not exist in the training data. Examples of these rare cases include a very dilated IVC with poor collapsibility due to heart failure or a very small IVC with complete collapsibility due to dehydration. Another “unknown” cluster may be IVC images that appear to collapse but represent artifacts in which the image appears to go out of plane of the ultrasound beam due to respiration movement.

#### Image analysis: IVC region contouring

To obtain the contour of the IVC region, we applied a lightweight segmentation algorithm [22]. As compared to other segmentation algorithms, this algorithm has a smaller size and high inference speed (> 60 frames per seconds), enabling its use in resource-limited settings and on handheld devices. Further details along with a visualization of the segmentation algorithm can be found in Appendix C.

After segmenting IVC region, the segmented region was cleaned to remove any isolated and unneeded pixels and retains a closed region. To delineate the IVC contour, we used the Moore-Neighbor tracing algorithm modified by Jacob’s stopping criteria [23]. After performing automated IVC region segmentation and contour delineation, the delineated contour was then used to compute IVC thickness or diameter in all frames of a given clip including the end-diastolic (largest dIVC) and end-systolic (smallest dIVC) frames as described next.

#### Image analysis: automated cIVC tracking

The delineated region, which was generated as described above, is divided into equal segments (or sectors). To compute dIVC, we automatically generated the major axis of the sub-segment that is located approximately 2 cm proximal to the ostium of the right atrium. We then computed the Euclidean distance between the endpoints of the major axis. Finally, we converted the computed pixel distance into millimeters (mm) as described in Appendix D. The computed dIVC was used to construct the dIVC curve by plotting the values over frames followed by applying a Savitzky-Golay filter [24] to obtain a smoothed dIVC curve. From this curve, the absolute maximum value (highest peak) of this measurement and the absolute minimum value (lowest valley) can be easily detected and used to measure the collapsibility percentage or cIVC. IVC collapsibility (cIVC) was computed based on the difference between the maximum peak and minimum valley in the dIVC curve. Specifically, cIVC is calculated as:

1 $$\begin{aligned} cIVC = \frac{dIVC_{max} - dIVC_{min}}{dIVC_{max}} \times 100\% \end{aligned}$$

#### Image analysis: automated RAP estimation

After the automated cIVC analysis, RAP is computed using the automatically generated dIVC and cIVC values based on two different criteria, namely ASE Criterion and NIH Criterion as detailed in Table 1.

*ASE Criterion* This criterion follows ASE guidelines [4, 5] for classifying RAP into 3 classes: 3, 8, and 15 mmHg based on dIVC and cIVC. A RAP of 3 mmHg is considered a normal or low pressure, indicating that the heart is functioning normally and there is no excessive pressure in the atrium; a RAP of 8 mmHg is considered slightly elevated, which can be caused by a variety of conditions such as heart failure, pulmonary hypertension, or fluid overload; a RAP of 15 mmHg or higher is considered severely elevated, which can indicate more severe heart failure, pulmonary embolism, or other serious cardiac conditions.

*NIH Criterion* This criterion follows a site-specific (NIH) guideline for classifying RAP as: 5, 10, 15, and 20 mmHg based on dIVC and cIVC. A RAP of 5 mmHg is considered a normal or low pressure; a RAP of 10 mm Hg is considered slightly elevated; a RAP of 15 mmHg would be considered moderately elevated; and a RAP of 20 mm Hg or higher would be considered severely elevated. Note that this criterion (NIH) has four RAP categories while the ASE criterion has three RAP categories due to historic precedents in this NIH echo lab.

**Table 1:** Different criteria (ASE and NIH) for RAP estimation

dIVC	cIVC	RAP (ASE Criterion)	RAP (NIH Criterion)
$\le$ 21 mm	> 50%	3 mmHg	5 mmHg
$\le$ 21 mm	< 50%	8 mmHg	10 mmHg
${>}$ 21 mm	> 50%	15 mmHg	15 mmHg
${>}$ 21 mm	< 50%	15 mmHg	20 mmHg

### Statistical analysis

Data are expressed as mean ± standard deviation (SD) unless specified. All dIVC measurements are considered continuous variables while cIVC^1^ and RAP values are considered categorical data. The Shapiro–Wilk test was used to test for the normality of IVC distribution. Automated versus manual reference measurements of IVC were compared using two-tailed, paired student’s t-test (or Mann-Whitney U-test if not normal), and a chi-square test was used for cIVC and RAP comparisons.

Additionally, Pearson correlation coefficient [25] and Bland-Altman [26] analyses were performed to assess the agreement between the automated dIVC measurements and those estimated by experts. To assess the agreement between the manual and automated RAP measurements, we used the confusion matrix, also known as a contingency table for categorical comparison. Various statistics were computed based on the values in the confusion matrix, such as macro accuracy, sensitivity, specificity, and f1-score to further evaluate how well the automated RAP values agree with the manual reference values.

**Table 2:** Demographic and clinical information for study participants

	Mean	SD	Max	Min
Age (year)	45	18	93	17
Height (m)	1.69	0.14	2.09	0.68
Weight (kg)	76.99	24.70	154.00	21.00
BMI (kg/$\text {m}^2$)	27.51	15.55	245.24	9.92
Systolic Blood Pressure (SBP), (mmHg)	122	17	210	76
Diastolic Blood Pressure (DBP), (mmHg)	69	12	120	32
Heart Rate (bpm)	80	18	142	45

## Results

Our dataset includes a similar distribution of male (128, $\approx$ 50%) and female (127, $\approx$ 50%) patients. The mean age was 44.99 ± 17.84 years. The mean weight and height were 76.99 ± 24.70 kg and 1.69 ± 0.14 cm, respectively. The mean body mass index (BMI) was 27.51 ± 15.55 kg/m2. The patients in our study had different clinical conditions including autoinflammatory disease, breast cancer, bronchiectasis, carcinoid, and SCD. The racial distribution is as follows: White ($\approx$ 66%), Hispanic ($\approx$ 11%), black or African American ($\approx$ 16%), Asian ($\approx$ 5%), and Mixed ($\approx$ 2%). Table 2 summarizes the demographic and clinical information for the study participants.

### Automated IVC retrieval and quality assessment

The downstream goal of IVC quantification requires accurate selection of individual subcostal IVC view from other views in each echocardiography study. Although others have previously published approaches in this area [14, 21, 27], our pipeline includes an automated stage to distinguish the IVC view from other echocardiographic views as well as subclasses of IVC views (e.g., view with artifact or very dilated IVC) with an accuracy of 0.97, a precision 0.96, a sensitivity of 0.97, and f-1 score of 0.96.

**Fig. 2:**
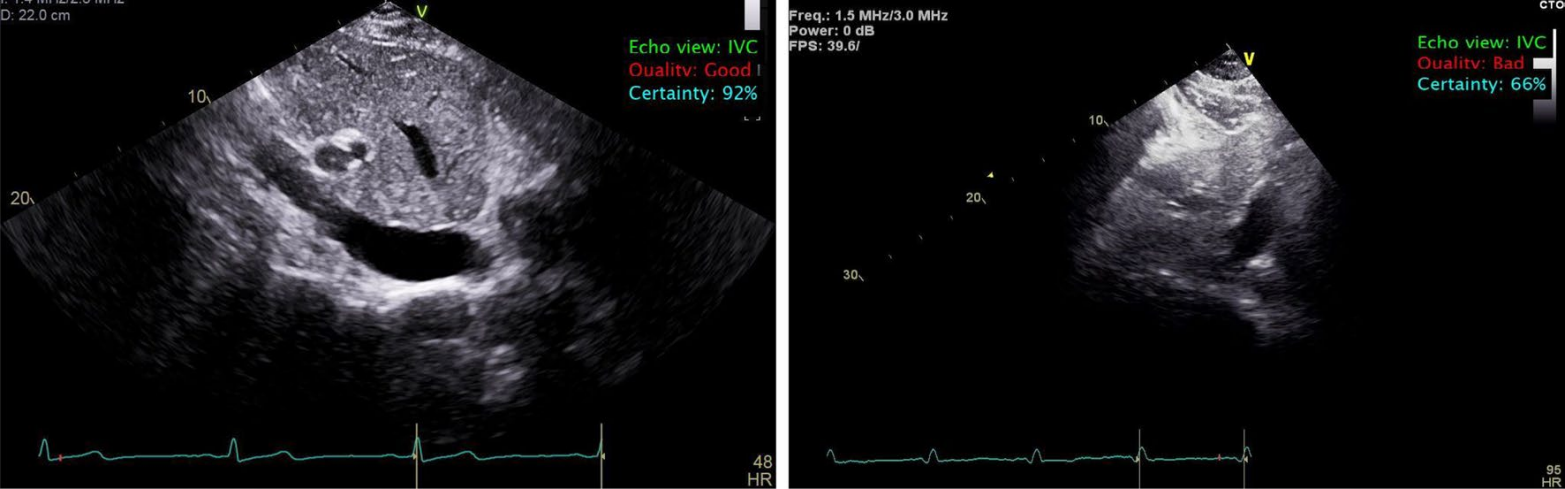
Left: example of good quality IVC view retrieved automatically from a set of other views; right: example of IVC view retrieved automatically from a set of other views and labeled as unusable (bad quality) as the IVC’s boundary is not clear

Another important step prior to automated IVC quantification is the quality assessment of the view. Several studies (e.g., [28]) reported that the accurate analysis of echocardiography is hugely dependent on the quality of the images, and that poor-quality images impair echocardiography quantifications. Therefore, we utilized a lightweight algorithm for assessing the quality of the IVC image prior to boundary delineation and thickness quantification. Our quality assessment algorithm achieved an accuracy of 0.94 ± 0.10, a precision of 0.94 ± 0.04, a sensitivity of 0.95 ± 0.09, and f-1 score of 0.95 ± 0.06. Figure 2 shows examples of echocardiography images classified as IVC with good and bad quality.

### Differences between manual and automated segmentation

To assess the differences between the manual and automated IVC region segmentation, we used intersection over union (IoU) [29] and dice similarity coefficient (DSC) [29]. IoU and DSC are measures of overlap between two sets of data and can be used to quantify the similarity or difference between manual and automated regions. The IoU is calculated as the intersection of the two regions divided by the union of the two regions while the DSC is calculated as twice the intersection of the two regions divided by the sum of the two regions. A high IoU or DSC indicates a strong agreement between the manual and automated regions, while a low score indicates disagreement or differences between the two regions.

**Fig. 3:**
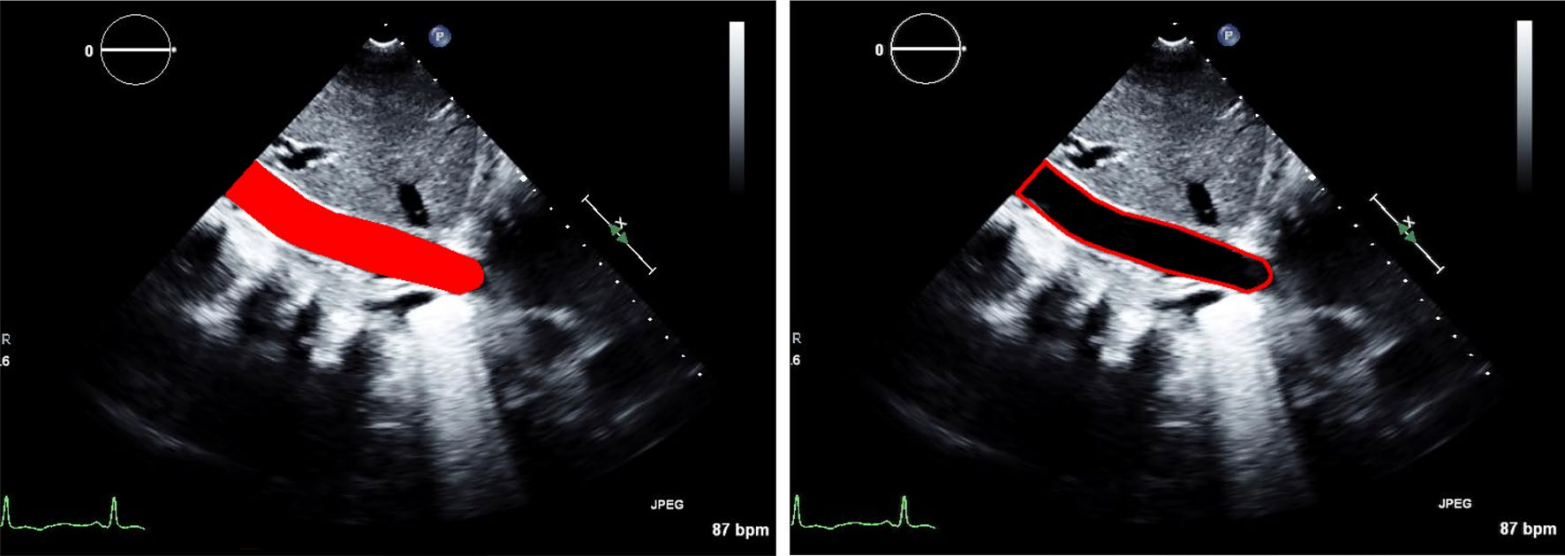
Left: the automatically generated mask overlayed on the original image; right: the contour of the overlayed mask

Our segmentation algorithm achieved excellent performance segmenting the IVC region with an IoU score of 0.96 ± 0.03 and a DSC score of 0.98 ± 0.05. In addition, our lightweight segmentation algorithm achieved an inference speed of > 60 frames per second (FPS). Figure 3 shows an example of the automatically segmented region along with the automatically generated contour.

### dIVC and cIVC tracking

**Fig. 4:**
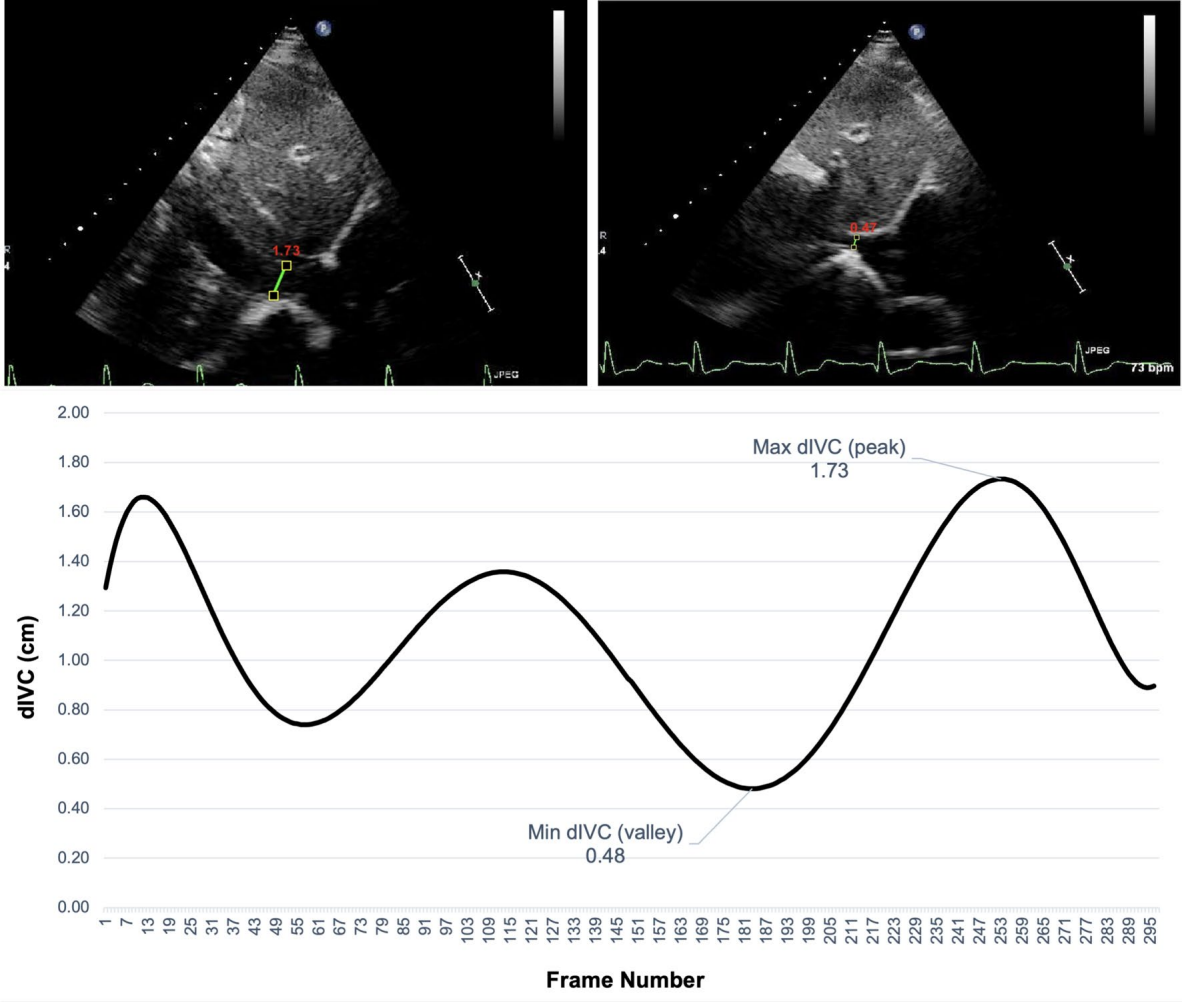
Top left: automated dIVC in frame 253 of a given video with a dIVC of 1.73 cm (maximum dIVC); top right: automated dIVC in frame 183 of a given video with a dIVC of 0.475 cm (minimum dIVC); bottom: dIVC in each frame of a given clip, where the peaks indicate maximum dIVC and valleys indicate minimum dIVC

The automatically delineated region in each frame is used to derive dIVC by measuring IVC diameter 2 cm from the junction of the right atrium. As this dIVC calculation is performed in each frame, the calculation of IVCs diameter was performed over frames. Figure 4 shows an example of the absolute maximum and minimum dIVC as well as dIVC curve over frames.

**Fig. 5:**
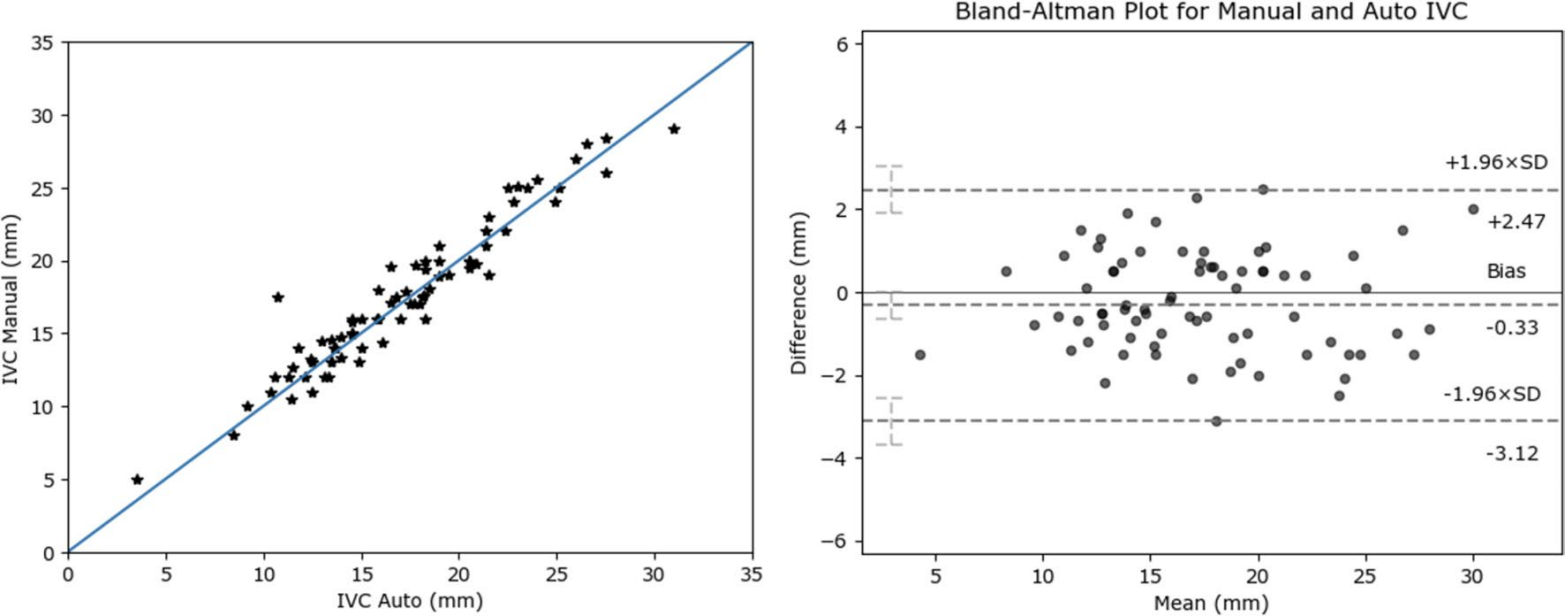
Correlation and B &A plots between automated and human dIVC (at 2 cm location)

For quantitative comparison of the automated versus manual dIVC measurements, the t-test shows there is no significant difference between the two groups (*p* = 0.70). To assess the agreement between the manual and automated dIVC, we used Pearson correlation and Bland-Altman plots. Figure 5 shows an excellent agreement between the automated dIVC measurements and those estimated by experts based on correlation (r = 0.96) and Bland-Altman plots. These results suggest that the automated method is accurate and allows assessing IVC diameter in each frame.

To further evaluate the automated method, we performed a variability analysis of measuring dIVC at different locations. Our results showed a strong agreement at 3 cm location (r = 0.95) as well as at 1 cm location (r = 0.87) with the manual reference standard. It is important to note that our automated algorithm is capable of measuring dIVC at various other spatial locations, including locations at the IVC-right atrium junction and locations 4 cm and 5 cm caudal to the junction. This feature enables the algorithm to perform variational analysis at different locations and time points, resulting in more reliable measurements. However, for this study, we only compared the manual and automated dIVC measurements at 2 cm from the right atrium junction since manual measurements were only available at that location.

For the subsequent comparison of cIVC, the manual and automated values were based off the dIVC measured at 2 cm from the right atrium junction since manual measurements were performed at that location. Recall that cIVC is estimated by plugging $dIVC_{max}$ and $dIVC_{min}$ into Eq. 1. For the cIVC comparison, the chi-square test showed that there is a significant association between the automated and manual estimates ($p<0.01$). After the automated dIVC and cIVC measurements were derived, they were used to generate the automated RAP estimates.

### Automated versus manual RAP estimation

To compare the automated and manual RAP values, the chi-square test shows there was a significant association between the manual versus automated RAP estimates by both ASE Criterion ($p<0.01$) as well as by NIH Criterion ($p<0.01$).

**Fig. 6:**
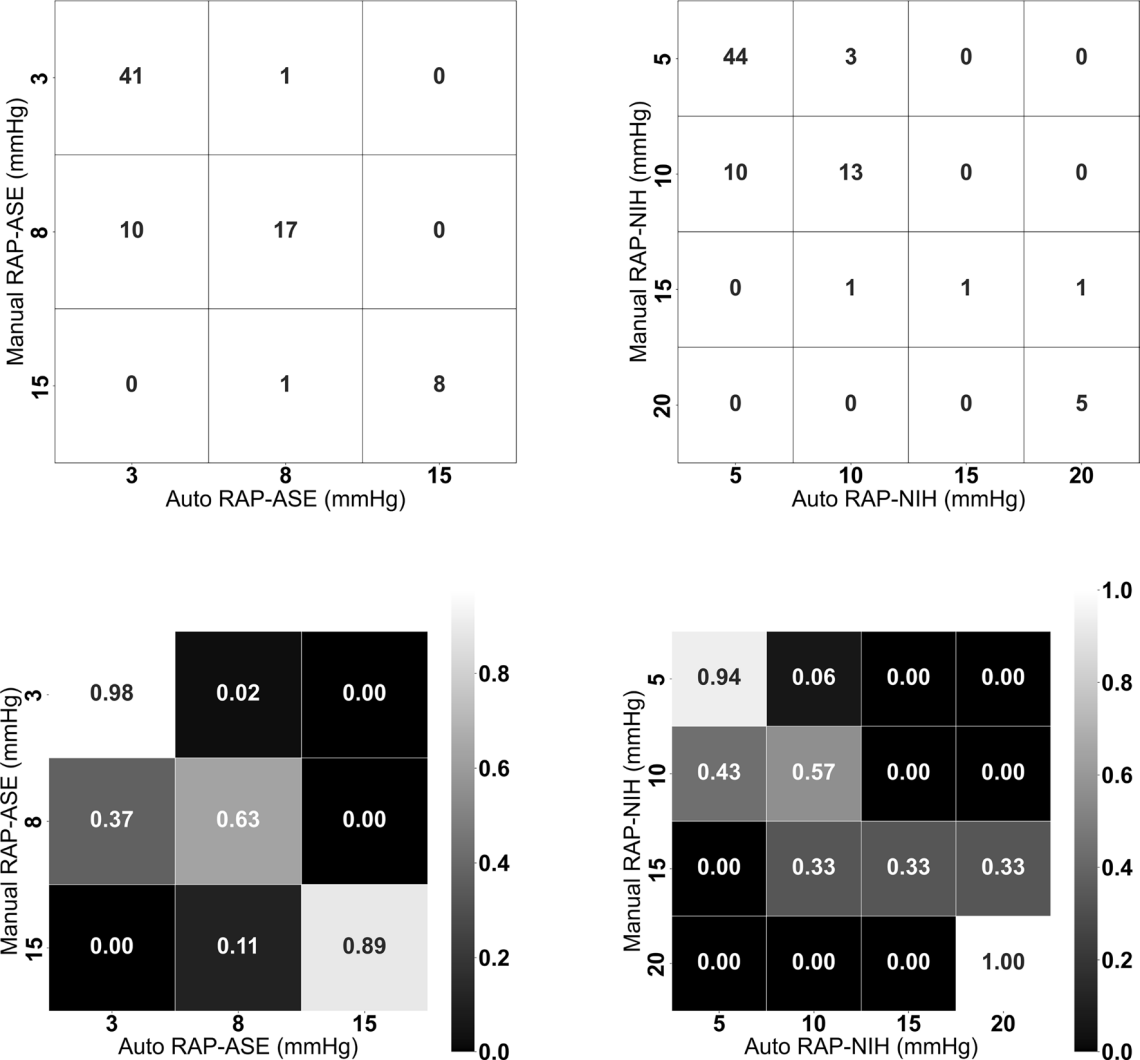
First row: non-normalized confusion matrices for automated RAP estimation using ASE Criterion (left) and NIH Criterion (right). Second row: normalized confusion matrices for automated RAP estimation using ASE Criterion (left) and NIH Criterion (right). The normalized confusion matrix is generated by dividing each value in the confusion matrix by the sum of the corresponding row

Figure 6 shows the confusion matrices for RAP estimates by both ASE and NIH criteria. From the matrices, we see a strong to moderate agreement for both criteria. The accuracy, precision, recall, and f-score for both ASE and NIH are presented in Table 3. Based on the findings from the table and corresponding figure, it appears that ASE Criterion slightly outperforms NIH Criterion. One possible explanation for this observation is that ASE Criterion has fewer classes (3, 8, and 15 mmHg) than NIH Criterion (5, 10, 15, and 20 mmHg), which could impact the performance of individual classes and consequently affect the overall performance. For example, the performance of RAP value of 10 is lower than other RAP values, impacting the overall performance of NIH Criterion. Despite the difference in the performance between ASE Criterion and NIH Criterion, both criteria achieved promising results and show that our AI-based method can be used to estimate RAP values reliably. It is important to note that our study primarily assessed the automated system’s ability to estimate RAP values using dIVC and cIVS. We have not yet compared these automated estimations to the gold-standard invasive RAP measurements. Such a comparison is planned for our subsequent research.

**Table 3:** Performance of automated RAP estimation using ASE and NIH criteria; the macro* accuracy of ASE Criterion is 0.90 and the macro accuracy of NIH Criterion is 0.85

	ASE RAP Guidelines			NIH (site-specific) Guidelines			
	3mmHg	8mmHg	15mmHg	5mmHg	10mmHg	15mmHg	20mmHg
Precision	0.80	0.89	1.00	0.81	0.76	1.00	0.83
Recall	0.98	0.63	0.89	0.94	0.57	0.33	1.00
F-score	0.88	0.74	0.94	0.87	0.65	0.50	0.91

*Macro accuracy treats all classes equally

## Discussion

This work presents an AI-system for the automation of dIVC, cIVC, and the subsequent estimation of RAP values. Our results show a strong agreement between automated measurements and those determined by human experts. Unlike current practices and existing automated methods, which predominantly compute dIVC in specified frames, our multi-stage AI system offers comprehensive echocardiography analysis in the open-world with minimal computational overhead. The system’s performance is robust, achieving a Pearson correlation coefficient of 0.96 and an F1-score of 0.85, outshining other referenced studies in these metrics.

Comparing our approach to the literature, in [15], a semi-automated LSTM-based architecture was trained on 220 videos, yielding moderate agreement (Fleiss’ kappa, *k*= 0.45) with expert IVC values. The subsequent work of the same authors, [16], employed this method to predict fluid responsiveness, achieving an AUC-ROC of 0.70 with 175 critically ill patients. In [17], Mesin et al. applied an edge-tracking and machine learning method to a dataset of 170 patients with specific heart-related conditions; the proposed method achieved moderate performance with an accuracy of 71% (SVM).

In contrast, our multi-stage AI-system covers the entire echocardiography analysis spectrum, from echo view selection to quantification. Further, our system takes into account challenges such as computational resource demands and data distribution shifts, incorporating efficient algorithms and adapting to open-world data changes. Additionally, a key strength of our system is its speed. Our algorithm not only facilitates echo view selection, quality assessment, and boundary tracing but also completes a comprehensive end-to-end echo analysis and quantifies parameters such as dIVC, CIVC, and RAP in less than a second (800ms on average). This streamlined efficiency is particularly beneficial in busy clinical settings. For context, we consulted an experienced cardiac sonographer from our echo lab who estimated that a visual assessment of dIVC takes around 3 s, nearly three times longer than our system’s full analysis. The manual measurement of dIVC takes even more time-consuming, averaging between 6 to 7 s, which is roughly 6 to 7 times lengthier than our automation. This automation could encourage the adoption of quantitative measurements in clinical settings. As highlighted in [30], the extended duration required for manual tracing of the cardiac borders has perpetuated the reliance on visual assessments in busy echocardiographic laboratories. Our innovation, therefore, represents a potential shift towards faster and more efficient analyses.

In addition to efficiency, our automated algorithm performs temporal analysis of dIVC over all video frames and at different sites. The analysis of all frames could provide information about temporal changes during respiration over multiple cardiac cycles. The temporal analysis feature of our system could also motivate sonographers to record longer echocardiography spans, potentially shedding light on respiratory changes’ impact on cardiac functions. In addition to the temporal analysis of dIVC and cIVC, our work investigated the automated estimation of noninvasive RAP measurements using two criteria, namely ASE criterion and NIH criterion. However, this work can be extended to estimate the gold-standard and noninvasive RAP measurement. Further, it could be extended to include other criteria and guidelines. There are several reasons for updating or customizing RAP estimation guidelines and recommendations to the specific resources and requirements of each organization or association. These reasons include differences in healthcare systems, new research findings, and variations in the patient populations. In the future, we plan to 1) evaluate the proposed system in estimating the gold-standard invasive RAP measurement, and 2) develop an automated algorithm that enables cardiologists to choose the best-suited RAP estimation criterion based on patient characteristics, and compare the results obtained from different guidelines to enhance accuracy, flexibility, and standardization of care.

Nonetheless, the present study was constrained by some limitations. First, it was conducted at a single center and the study cohort was made up of patients who underwent echocardiography examination for any reason, which might impact the results when the system is applied to patient groups with specific diseases such as pulmonary hypertension, cardiac tamponade, fluid overload, or patients with critically ill conditions.

Second, while our AI algorithm provides consistent results – that is, it can reproduce the exact same measurements for dIVC, cIVC, and RAP in each run – our current study did not comprehensively assess inter-observer and intra-observer variability. Such an assessment would be crucial in determining the reproducibility, which can be influenced by human’s variability. Existing literature has underscored the potential benefits of AI-driven reproducibility in echocardiography. For instance, Nolan et al. [31] reported that automated systems tend to exhibit consistent measurements across different cases, largely eliminating the variability often encountered in manual measurements. Such findings suggest that AI-driven methods, like ours, could offer a notable advantage in enhancing consistency in echocardiographic assessments. However, we recognize the importance of comparing the consistent output of our algorithm against the potential variability seen in manual measurements, and we realize the importance of conducting reproducibility analysis.

Finally, a pivotal limitation in our study is its failure to compare automated RAP values against the gold-standard invasive RAP measurements. While our system demonstrated reliability in IVC measurements relative to the manual method, its capability for direct RAP estimation using the gold standard approach remains unvalidated. In acknowledging this limitation, our next immediate step will involve validating the performance of our system against the invasive gold-standard RAP measurements. In our continued research, we also aim to evaluate our proposed system across different centers, conduct thorough intra- and inter-observer variability evaluations, compare the time required for automated and manual IVC analysis, and provide a more nuanced comparison between automated and manual IVC measurements across different spatial locations.

## Conclusion

We present an AI-based system dedicated to automating dIVC and cIVC measurements, which has the potential to refine the current clinical practice of IVC analysis. Specifically, our solution provides a fully automated, cost-effective, and quantitative tool for dIVC and cIVC analysis that could be used in clinical settings and point-of-care testing. Moreover, it offers the capability to conduct variational analysis of dIVC across diverse spatial locations and temporal points, thereby ensuring more consistent measurements. While there may be potential implications for RAP estimations, the primary intent of our system is to augment the current practice of IVC analysis. Such improvements could potentially lead to better clinical decision-making and improved patient outcomes.

## Figures and Tables

**Fig. 7:**
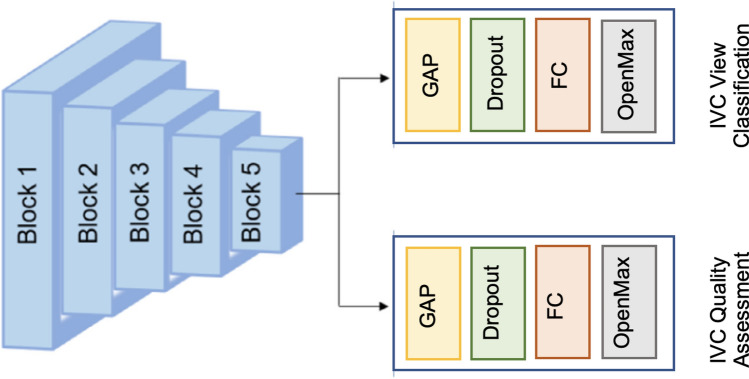
Overview of IVC retrieval (quality assessment and view classification) stage. Each block represents MobileNetV2-s residual blocks. GAP and FC represent the global average pooling layer and fully connected layer, respectively. OpenMax is a replacement for the SoftMax function that enables open-world learning

**Fig. 8:**
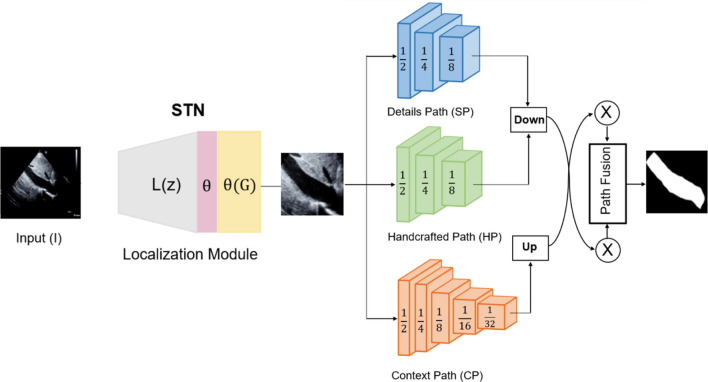
TaNet for IVC region segmentation. TaNet has two main components: STN for IVC region localization and segmentation with 3 pathways, spatial (detail) path (SP), handcrafted path (HP), global or context path (GP). STN focuses the segmentation attention on the cardiac region. The numbers in the segmentation cubes are the size ratios to the resolution of the input. *L*(*z*), $\theta$, and $\theta (G)$ are STN parameters

## Appendix A two-head classification algorithm

The image retrieval algorithm retrieves a specific view (i.e., IVC view or sub-views) with acceptable quality. As shown in Fig. 7, our algorithm utilizes a lightweight model with a shared encoder and two heads. The shared encoder includes five inverted MobileNetV2-s [32] residual blocks. The two heads perform view classification and quality assessment and are configured to operate with the shared encoder. Each head has the following layers: a Global Average Pooling (GAP) layer, a Dropout layer, Fully Connected (FC) layer, and an OpenMax layer.

We fine-tuned each head along with its shared encoder as follows. First, we initialized MobileNetV2-s encoder with the echo-specific weights (transfer learning) followed by fine-tuning the echo-specific encoder and the view classification layers using the datasets presented in Section 2.1. The view classification head is fine tuned to minimize the categorical cross entropy (CCE) loss using stochastic gradient descent (SGD) optimizer. We used a batch size of 32, for 32 epochs, and an initial learning rate of $1 \times 10^{-3}$. This head classifies a given echo as IVC, other (e.g., PLAX, A4C, Doppler), or unknown. Similar to the view classification head, the quality assessment head is fine-tuned to minimize binary cross entropy (BCE) loss using SGD optimizer with a batch size of 16, for 32 epochs, and an initial learning rate of $1 \times 10^{-3}$. This head classifies a given echo image/video as good quality or bad quality. In clinical practice, echocardiographers visually identify echo views and manually exclude low-quality echoes as they lead to inaccurate measurements. Since our image retrieval component is lightweight, it enables echo view classification and quality assessment in clinical practice.

## Appendix B open-world active learning

Although machine learning-based algorithms achieved high performance on several visual recognition tasks including image classification and segmentation [33], most of these algorithms are designed to only learn images belonging to a predefined set of classes given before training. In a closed world setting, we assume [33] that both $D_{train}$ and $D_{test}$ are drawn from the same distribution, and the classifier is trained using $D_{train}$ to minimize an empirical loss function (e.g., cross-entropy). This loss function is optimized to discriminate between different known classes. Finally, the trained closed world classifier is tested using $D_{test}$ to label a new image as one of the known classes in *Y*. Although the closed world assumption holds in several applications, many real-world applications are dynamic and open containing examples from classes that might not appear in training [34]. Typically, a closed world classifier would classify an unseen or unknown example as one of the known classes. Since the cost of randomly misclassifying an unseen image to a known class can be high, especially in clinical practice, there is a need to design robust classifiers for open world settings.

In such settings, the classifier is still trained using $D_{train}$. However, $D_{test}$ has a set (*Y*) containing predetermined classes as well as unknown classes; i.e., $y_i$ $\in$ *Y* = 1, 2,..*C*, $C + 1$), where *C* represents the number of known classes and $C+1$ represents the new class. Like the close world classifier, the open world classifier is trained to minimize a loss function with an overall aim to recognize known classes and classify unknown classes as $C+1$.

Open world learning has been integrated into convolutional neural networks (CNNs) to create robust deep open classification (DOC) models [35, 36]. In [36], Shu et al. integrated open world learning into CNNs by employing a 1-vs.-rest layer. This layer uses Sigmoid activation functions and Gaussian fitting to classify known classes while rejecting unknown ones. It has *N* Sigmoid functions for *N* known classes; it then rejects unseen classes based on thresholding (*t*). Although DOC has been widely used for open world deep learning classification, other methods have been used. For example, a simpler method is thresholding on the Softmax output; i.e., a given input image is labeled as unknown if none of the classes reaches a predetermined threshold. The performance of this approach is sensitive to the used threshold, which must be estimated empirically from the training dataset.

Another method that has been widely used to integrate open world learning into deep learning models is OpenMax [20]. In this method, the traditional Softmax layer is extended to predict unknown classes using the likelihood of failure and the concept of meta-recognition [20]. To estimate if the input is unknown or ”far" from known classes, the scores from the penultimate layer of convolutional neural networks (i.e., fully connected layer) are used. Then, inputs that are far (in terms of distribution) from known classes are classified as unknown or rejected.

In this work, we replaced the Softmax function with the OpenMax [20] function. Our open-world IVC retrieval algorithm works as follows. During each iteration, the classifier either detects a specific echocardiography view as IVC or labels it as unknown. Then, unknown views are grouped into clusters or groups (based on their similarity) to be labeled by a human expert before passing the newly labeled clusters/classes for a model update. This process of labeling unknown images that are previously unseen during the model’s training, clustering them, and obtaining human feedback is called open-world active learning. We refer the reader to [21] for further details about our open-world active learning algorithm.

## Appendix C IVC segmentation algorithm

Figure 8 depicts our TaNet for cardiac region segmentation. Our TaNet algorithm, which was proposed in [22], localizes the region of interest (i.e., IVC) using a localization algorithm and then uses three pathways for learning rich textural, low-level, and context features. Current convolutional neural networks (CNNs) operate on the whole image and are limited by the spatial invariance of input data. The traditional approach for handling these issues involves using separate models for spatial transformation and localization; i.e., special object detection models are used to locate the region of interest before segmenting that region. Jaderberg et al. [37] proposed a more efficient transformation network, called Spatial Transformer Network (STN), for applying spatial transformations (e.g., scaling, translation, attention/detection) to the input image or feature map without additional training supervision. STN is a plug-and-play module that can be easily inserted into existing CNNs. It is also differentiable in the sense that it computes the derivative of the transformations within the module, which allows learning the loss gradients with respect to the module parameters.

In medical images, it is common that the target region occupies a relatively small portion of the image. Hence, considering the entire image for segmentation would add noise caused by irrelevant regions. In this work, we use STN for focusing the attention of the segmentation/contouring algorithm on the IVC region while suppressing irrelevant regions in the background.

After focusing the attention on the IVC region using STN algorithm, we used the lightweight segmentation algorithm (see Fig. 8) to segment IVC from the background. The segmentation algorithm has three pathways: spatial or detail pathway (SP), handcrafted pathway (HP), and context pathway (CP). Each of these pathways extract a unique set of features as described next.

*Spatial Pathway (SP)* To extract rich low-level details (e.g., edges, color blobs) at a low computational cost, a shallow pathway that has three layers with high channel capacity is adopted. Specifically, we used three blocks, each containing a $3\times 3$ convolutional layer with stride of 2 followed by batch normalization and ReLU activation. The number of filters in the first, second, and third blocks are 64, and 128, respectively.

*Handcrafted pathway (HP)* Depending on the medical imaging modality and the application, the standard convolutional kernels can be replaced by handcrafted-based kernels to extract a unique set of statistical, geometrical, or textural features. As compared to the handcrafted-based methods, the main strength of deep learning is its ability to learn features at different levels of abstraction, which allows learning complex functions that map the input to the output. However, these complex functions may be generic. On the other hand, hand-crafted descriptors or kernels are designed to extract specific features (e.g., textural, geometric) that may be different from the ones extracted by deep learning models (e.g., edges, color blobs). For example, the textural features (e.g., Local binary pattern [LBP]) have strong ability to differentiate small differences in texture and topography especially at the boundaries between complex regions with challenging separations.

In this work, we integrated handcrafted kernels into CNN learning. Similar to the spatial pathway (SP), we add a handcrafted pathway (HP) with three convolutional blocks, but replace the standard convolutional filters with LBP filters. These LBP-encoded convolutional kernels are used to extract rich texture features from the echo images. Each LBP block has a layer with fixed anchor weights (m) followed by a second layer with learnable convolutional filters of size $1\times 1$. We generated the anchor weights stochastically with different ranges of sparsity.

*Context Path (CP)* The last branch is used for fast down-sampling of the feature map of the input image to obtain a sufficiently scoped receptive field for encoding high-level context information. Subsequently, a GAP layer is attached to the tail of the lightweight model to provide the maximum receptive field with global context information. In segmentation, the network analyzes the feature map of the input image at different receptive fields. The receptive field indicates the extent of the scope of input data a neuron or unit within a layer can be exposed to and is defined by the filter size of a layer within a convolution neural network. Finally, the output of the global average pooling is up-sampled and combined with the output of other pathways.

*Path Fusion* In the last stage, the outputs of the three pathways are combined using a fusion module to obtain a weighted feature vector. In particular, the fusion module fuses the features from the three paths by first concatenating the pathways’ outputs and then using batch normalization to balance the different scales of the features. Then, the concatenated features are combined into a single feature vector. This feature vector is sent to a global pooling and followed by a convolutional layer (1x1), and a Sigmoid function is used to generate the weight vector. We refer the reader to [22] for further details about this segmentation network.

## Appendix D automated quantification

Instead of computing cardiac measurements in specific frames, the temporal analysis of measurements over frames has great applicability in clinical cardiology practice and research. It provides information about the mechanics of cardiac chambers and captures how they change over time. We present next the steps for computing dIVC from the segmented regions.

Prior to the delineation of IVC boundaries, we perform morphological cleaning to remove any isolated pixels and only keep the closed region of interest. Next, we compute the contour of the clean regions using Moore-Neighbor tracing algorithm modified by Jacob’s stopping criteria ([23]). Next, the delineated region is divided into equal segments (or sectors). These segments are then used to compute dIVC at different spatial locations.

To compute dIVC at 2 cm location, we find the major axis of the sub region that is located approximately 2 cm proximal to the ostium of RA. We then compute the Euclidean distance between the endpoints of the major axis. Finally, we convert the computed pixel distance into millimeters (mm) as follows:

D1 $$\begin{aligned} Pixel&= (25.4 \div dpi) \text { mm} \end{aligned}$$

D2 $$\begin{aligned} dIVC&= dIVC_{distance} \times Pixel \end{aligned}$$

where *dpi* is the dots per inch in a given clip. After computing *dIVC*, we construct the *dIVC* curve by plotting *dIVC* values over frames. Next, we use the Savitzky-Golay filter to obtain a smoothed *dIVC* curve. The smoothed curve is then used to compute RAP as follows. First, we compute cIVC based on the difference between the maximum peak and minimum valley in the *dIVC* curve:

D3 $$\begin{aligned} Collapsibility = \frac{dIVC_{peak} - dIVC_{valley}}{dIVC_{peak}} \times 100\% \end{aligned}$$

Finally, the RAP value is computed by plugging the IVC diameter and collapsibility values into the equation provided by ASE criterion or NIH criterion.

## Abbreviations

AI: Artificial intelligence
ML: Machine learning
IVC: Inferior vena cava
RAP: Right atrial pressure
dIVC: Diameter of inferior vena cava
cIVC: Collapsibility index of inferior vena cava
GAP: Global average pooling
LBP: Local binary pattern kernel
FC: Fully connected layer
IoU: Intersection over union
DSC: Dice similarity coefficient
FPS: Frames per second
LSTM: Long short-term memory
PLAX: Long axis view
A4C: Four-chamber view

## Footnotes

1 cIVC values > 50% are labeled as 1 (collapsibility present) and cIVC values < 50% are labeled as 0 (collapsibility absent).
